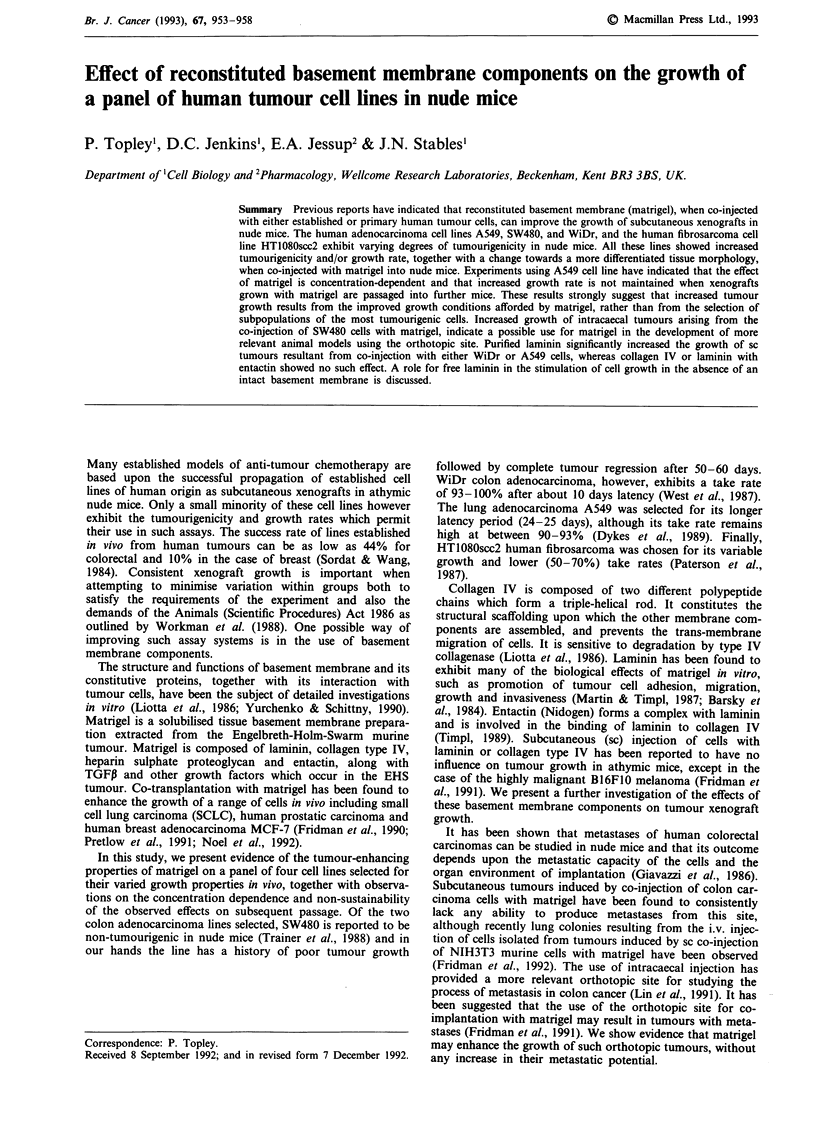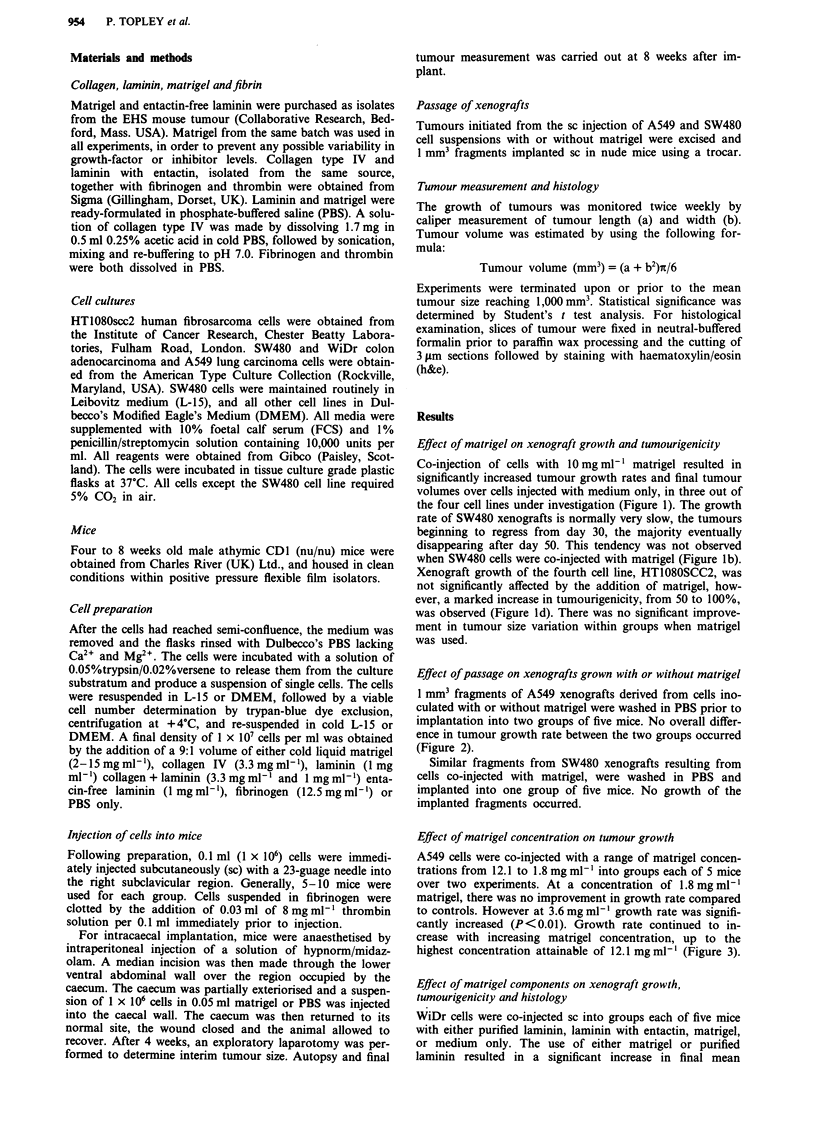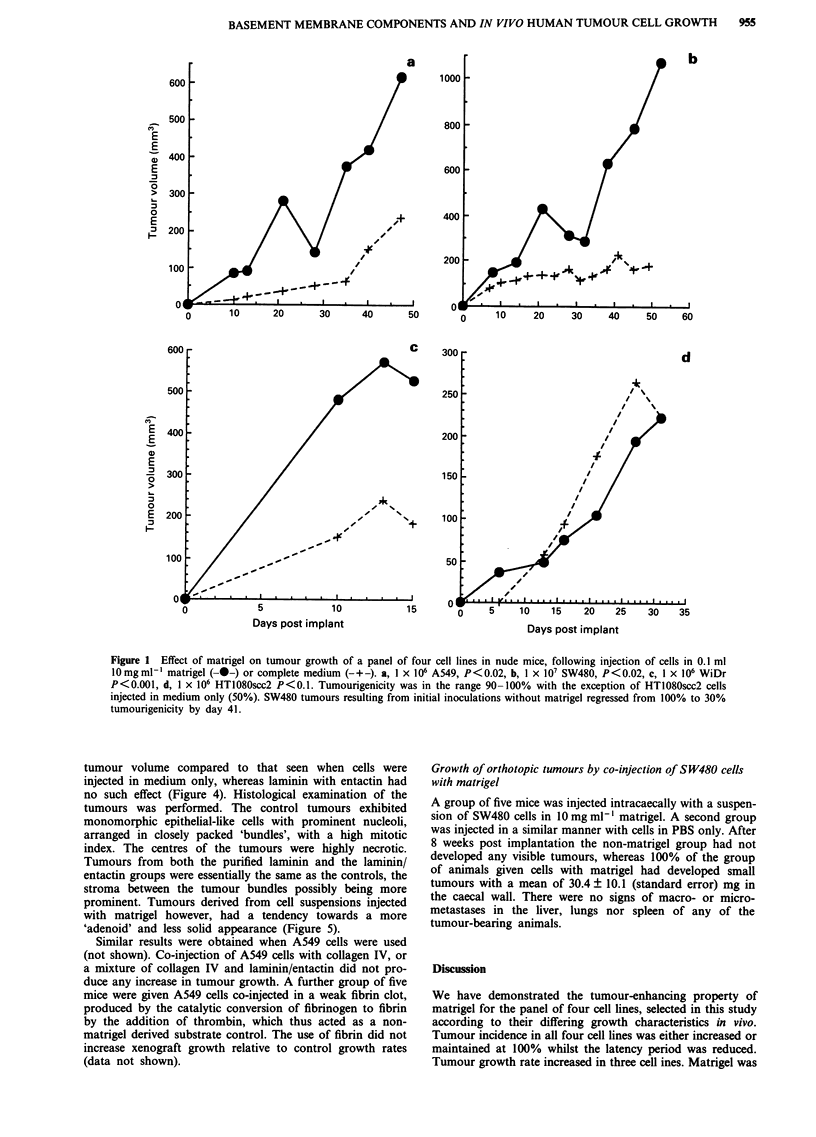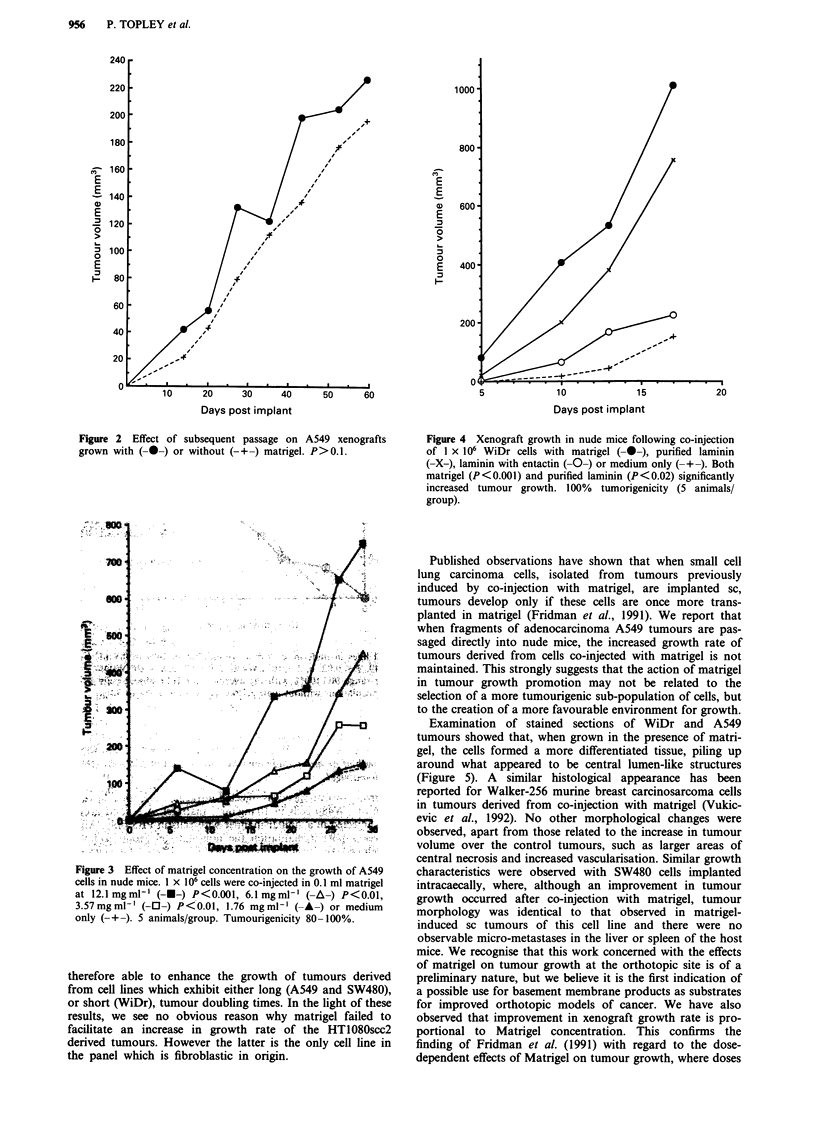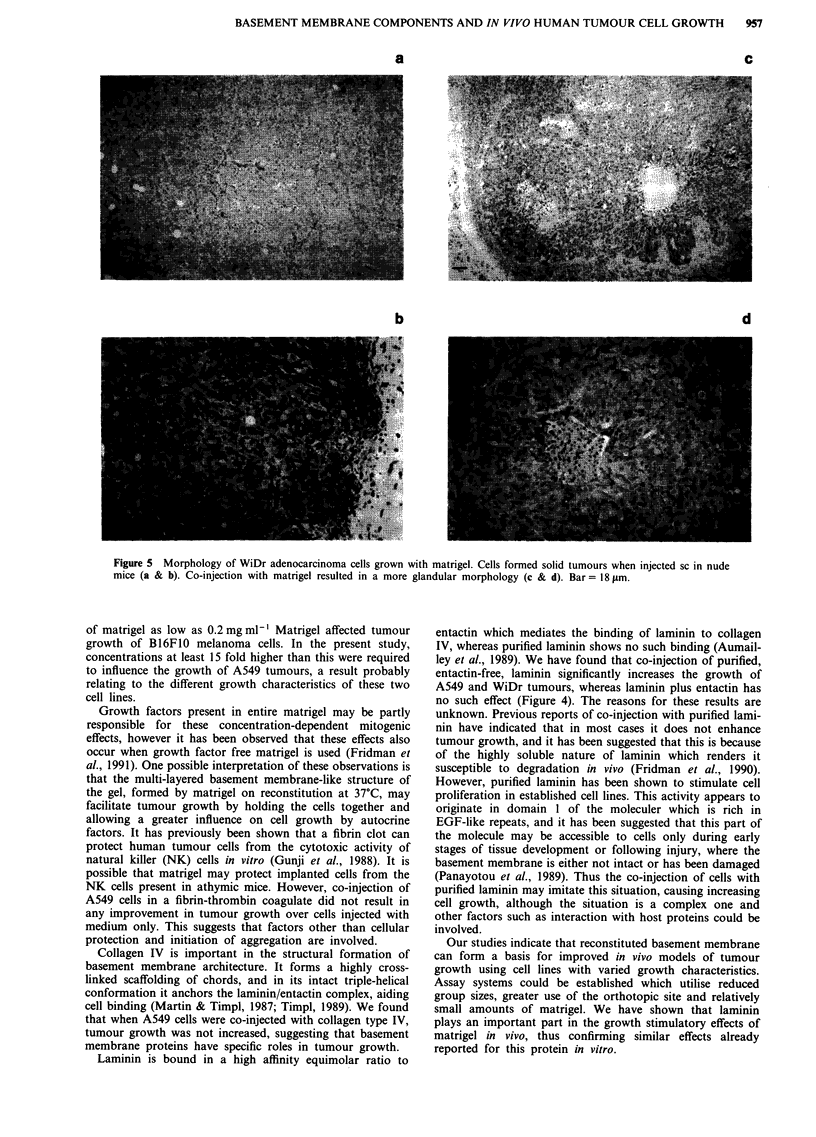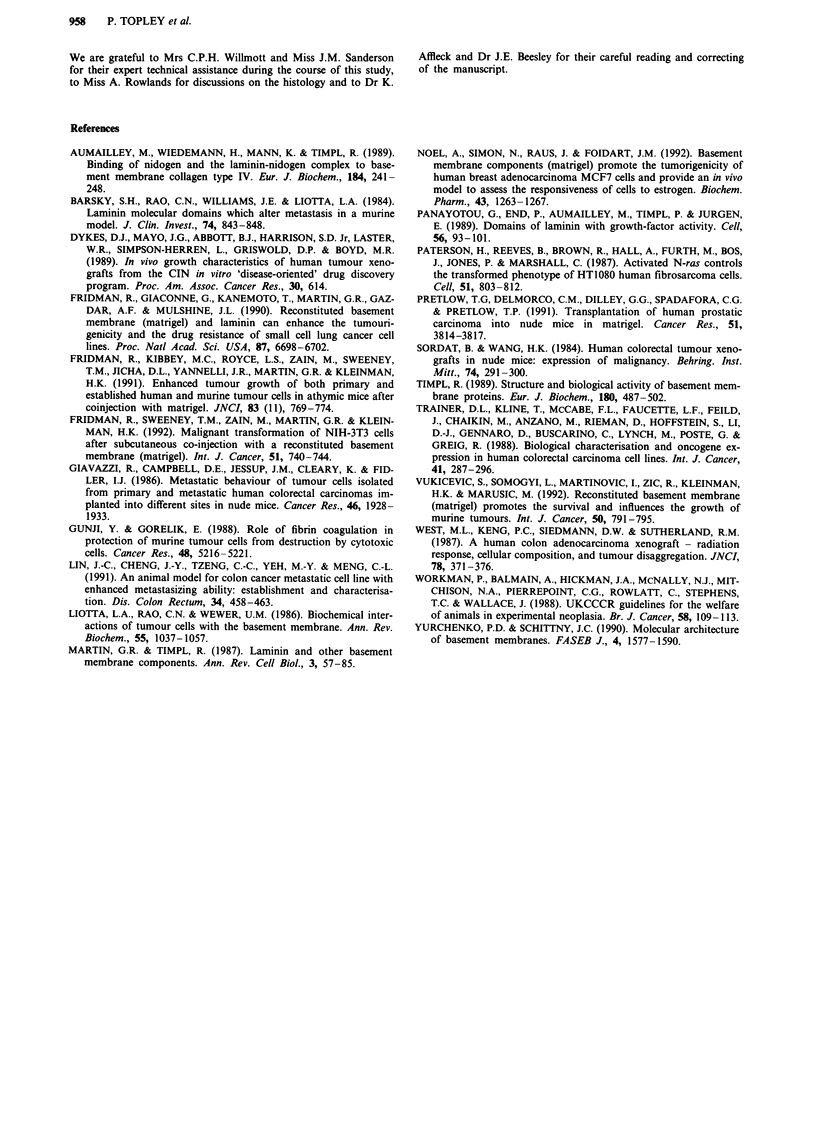# Effect of reconstituted basement membrane components on the growth of a panel of human tumour cell lines in nude mice.

**DOI:** 10.1038/bjc.1993.176

**Published:** 1993-05

**Authors:** P. Topley, D. C. Jenkins, E. A. Jessup, J. N. Stables

**Affiliations:** Department of Cell Biology, Wellcome Research Laboratories, Beckenham, Kent, UK.

## Abstract

**Images:**


Br. J. Cancer (1993), 67, 953–958 © Macmillan Press Ltd., 1993

### Effect of reconstituted basement membrane components on the growth of a panel of human tumour cell lines in nude mice

P. Topley[1], D.C. Jenkins[1], E.A. Jessup[2] & J.N. Stables[1]

*Department of [1]Cell Biology and [2]Pharmacology, Wellcome Research Laboratories, Beckenham, Kent BR3 3BS, UK.*

**Summary** Previous reports have indicated that reconstituted basement membrane (matrigel), when co-injected with either established or primary human tumour cells, can improve the growth of subcutaneous xenografts in nude mice. The human adenocarcinoma cell lines A549, SW480, and WiDr, and the human fibrosarcoma cell line HT1080scc2 exhibit varying degrees of tumourigenicity in nude mice. All these lines showed increased tumourigenicity and/or growth rate, together with a change towards a more differentiated tissue morphology, when co-injected with matrigel into nude mice. Experiments using A549 cell line have indicated that the effect of matrigel is concentration-dependent and that increased growth rate is not maintained when xenografts grown with matrigel are passaged into further mice. These results strongly suggest that increased tumour growth results from the improved growth conditions afforded by matrigel, rather than from the selection of subpopulations of the most tumourigenic cells. Increased growth of intracaecal tumours arising from the co-injection of SW480 cells with matrigel, indicate a possible use for matrigel in the development of more relevant animal models using the orthotopic site. Purified laminin significantly increased the growth of sc tumours resultant from co-injection with either WiDr or A549 cells, whereas collagen IV or laminin with entactin showed no such effect. A role for free laminin in the stimulation of cell growth in the absence of an intact basement membrane is discussed.

Many established models of anti-tumour chemotherapy are based upon the successful propagation of established cell lines of human origin as subcutaneous xenografts in athymic nude mice. Only a small minority of these cell lines however exhibit the tumourigenicity and growth rates which permit their use in such assays. The success rate of lines established *in vivo* from human tumours can be as low as 44% for colorectal and 10% in the case of breast (Sordat & Wang, 1984). Consistent xenograft growth is important when attempting to minimise variation within groups both to satisfy the requirements of the experiment and also the demands of the Animals (Scientific Procedures) Act 1986 as outlined by Workman *et al.* (1988). One possible way of improving such assay systems is in the use of basement membrane components.

The structure and functions of basement membrane and its constitutive proteins, together with its interaction with tumour cells, have been the subject of detailed investigations *in vitro* (Liotta *et al.*, 1986; Yurchenko & Schittny, 1990). Matrigel is a solubilised tissue basement membrane preparation extracted from the Engelbreth-Holm-Swarm murine tumour. Matrigel is composed of laminin, collagen type IV, heparin sulphate proteoglycan and entactin, along with TGF$\beta$ and other growth factors which occur in the EHS tumour. Co-transplantation with matrigel has been found to enhance the growth of a range of cells *in vivo* including small cell lung carcinoma (SCLC), human prostatic carcinoma and human breast adenocarcinoma MCF-7 (Fridman *et al.*, 1990; Pretlow *et al.*, 1991; Noel *et al.*, 1992).

In this study, we present evidence of the tumour-enhancing properties of matrigel on a panel of four cell lines selected for their varied growth properties *in vivo*, together with observations on the concentration dependence and non-sustainability of the observed effects on subsequent passage. Of the two colon adenocarcinoma lines selected, SW480 is reported to be non-tumourigenic in nude mice (Trainer *et al.*, 1988) and in our hands the line has a history of poor tumour growth followed by complete tumour regression after 50–60 days. WiDr colon adenocarcinoma, however, exhibits a take rate of 93–100% after about 10 days latency (West *et al.*, 1987). The lung adenocarcinoma A549 was selected for its longer latency period (24–25 days), although its take rate remains high at between 90–93% (Dykes *et al.*, 1989). Finally, HT1080scc2 human fibrosarcoma was chosen for its variable growth and lower (50–70%) take rates (Paterson *et al.*, 1987).

Collagen IV is composed of two different polypeptide chains which form a triple-helical rod. It constitutes the structural scaffolding upon which the other membrane components are assembled, and prevents the trans-membrane migration of cells. It is sensitive to degradation by type IV collagenase (Liotta *et al.*, 1986). Laminin has been found to exhibit many of the biological effects of matrigel *in vitro*, such as promotion of tumour cell adhesion, migration, growth and invasiveness (Martin & Timpl, 1987; Barsky *et al.*, 1984). Entactin (Nidogen) forms a complex with laminin and is involved in the binding of laminin to collagen IV (Timpl, 1989). Subcutaneous (sc) injection of cells with laminin or collagen type IV has been reported to have no influence on tumour growth in athymic mice, except in the case of the highly malignant B16F10 melanoma (Fridman *et al.*, 1991). We present a further investigation of the effects of these basement membrane components on tumour xenograft growth.

It has been shown that metastases of human colorectal carcinomas can be studied in nude mice and that its outcome depends upon the metastatic capacity of the cells and the organ environment of implantation (Giavazzi *et al.*, 1986). Subcutaneous tumours induced by co-injection of colon carcinoma cells with matrigel have been found to consistently lack any ability to produce metastases from this site, although recently lung colonies resulting from the i.v. injection of cells isolated from tumours induced by sc co-injection of NIH3T3 murine cells with matrigel have been observed (Fridman *et al.*, 1992). The use of intracaecal injection has provided a more relevant orthotopic site for studying the process of metastasis in colon cancer (Lin *et al.*, 1991). It has been suggested that the use of the orthotopic site for co-implantation with matrigel may result in tumours with metastases (Fridman *et al.*, 1991). We show evidence that matrigel may enhance the growth of such orthotopic tumours, without any increase in their metastatic potential.

Correspondence: P. Topley.
Received 8 September 1992; and in revised form 7 December 1992.

### Materials and methods

*Collagen, laminin, matrigel and fibrin*

Matrigel and entactin-free laminin were purchased as isolates from the EHS mouse tumour (Collaborative Research, Bedford, Mass. USA). Matrigel from the same batch was used in all experiments, in order to prevent any possible variability in growth-factor or inhibitor levels. Collagen type IV and laminin with entactin, isolated from the same source, together with fibrinogen and thrombin were obtained from Sigma (Gillingham, Dorset, UK). Laminin and matrigel were ready-formulated in phosphate-buffered saline (PBS). A solution of collagen type IV was made by dissolving 1.7 mg in 0.5 ml 0.25% acetic acid in cold PBS, followed by sonication, mixing and re-buffering to pH 7.0. Fibrinogen and thrombin were both dissolved in PBS.

*Cell cultures*

HT1080scc2 human fibrosarcoma cells were obtained from the Institute of Cancer Research, Chester Beatty Laboratories, Fulham Road, London. SW480 and WiDr colon adenocarcinoma and A549 lung carcinoma cells were obtained from the American Type Culture Collection (Rockville, Maryland, USA). SW480 cells were maintained routinely in Leibovitz medium (L-15), and all other cell lines in Dulbecco's Modified Eagle's Medium (DMEM). All media were supplemented with 10% foetal calf serum (FCS) and 1% penicillin/streptomycin solution containing 10,000 units per ml. All reagents were obtained from Gibco (Paisley, Scotland). The cells were incubated in tissue culture grade plastic flasks at 37°C. All cells except the SW480 cell line required 5% $CO_2$ in air.

*Mice*

Four to 8 weeks old male athymic CD1 (nu/nu) mice were obtained from Charles River (UK) Ltd., and housed in clean conditions within positive pressure flexible film isolators.

*Cell preparation*

After the cells had reached semi-confluence, the medium was removed and the flasks rinsed with Dulbecco's PBS lacking $Ca^{2+}$ and $Mg^{2+}$. The cells were incubated with a solution of 0.05%trypsin/0.02%versene to release them from the culture substratum and produce a suspension of single cells. The cells were resuspended in L-15 or DMEM, followed by a viable cell number determination by trypan-blue dye exclusion, centrifugation at +4°C, and re-suspended in cold L-15 or DMEM. A final density of $1 \times 10^7$ cells per ml was obtained by the addition of a 9:1 volume of either cold liquid matrigel (2–15 mg $ml^{-1}$), collagen IV (3.3 mg $ml^{-1}$), laminin (1 mg $ml^{-1}$) collagen + laminin (3.3 mg $ml^{-1}$ and 1 mg $ml^{-1}$) entacin-free laminin (1 mg $ml^{-1}$), fibrinogen (12.5 mg $ml^{-1}$) or PBS only.

*Injection of cells into mice*

Following preparation, 0.1 ml ($1 \times 10^6$) cells were immediately injected subcutaneously (sc) with a 23-guage needle into the right subclavicular region. Generally, 5–10 mice were used for each group. Cells suspended in fibrinogen were clotted by the addition of 0.03 ml of 8 mg $ml^{-1}$ thrombin solution per 0.1 ml immediately prior to injection.

For intracaecal implantation, mice were anaesthetised by intraperitoneal injection of a solution of hypnorm/midazolam. A median incision was then made through the lower ventral abdominal wall over the region occupied by the caecum. The caecum was partially exteriorised and a suspension of $1 \times 10^6$ cells in 0.05 ml matrigel or PBS was injected into the caecal wall. The caecum was then returned to its normal site, the wound closed and the animal allowed to recover. After 4 weeks, an exploratory laparotomy was performed to determine interim tumour size. Autopsy and final tumour measurement was carried out at 8 weeks after implant.

*Passage of xenografts*

Tumours initiated from the sc injection of A549 and SW480 cell suspensions with or without matrigel were excised and 1 $mm^3$ fragments implanted sc in nude mice using a trocar.

*Tumour measurement and histology*

The growth of tumours was monitored twice weekly by caliper measurement of tumour length (a) and width (b). Tumour volume was estimated by using the following formula:

$$\text{Tumour volume } (mm^3) = (a + b^2)\pi/6$$

Experiments were terminated upon or prior to the mean tumour size reaching 1,000 $mm^3$. Statistical significance was determined by Student's $t$ test analysis. For histological examination, slices of tumour were fixed in neutral-buffered formalin prior to paraffin wax processing and the cutting of 3 μm sections followed by staining with haematoxylin/eosin (h&e).

### Results

*Effect of matrigel on xenograft growth and tumourigenicity*

Co-injection of cells with 10 mg $ml^{-1}$ matrigel resulted in significantly increased tumour growth rates and final tumour volumes over cells injected with medium only, in three out of the four cell lines under investigation (Figure 1). The growth rate of SW480 xenografts is normally very slow, the tumours beginning to regress from day 30, the majority eventually disappearing after day 50. This tendency was not observed when SW480 cells were co-injected with matrigel (Figure 1b). Xenograft growth of the fourth cell line, HT1080SCC2, was not significantly affected by the addition of matrigel, however, a marked increase in tumourigenicity, from 50 to 100%, was observed (Figure 1d). There was no significant improvement in tumour size variation within groups when matrigel was used.

*Effect of passage on xenografts grown with or without matrigel*

1 $mm^3$ fragments of A549 xenografts derived from cells inoculated with or without matrigel were washed in PBS prior to implantation into two groups of five mice. No overall difference in tumour growth rate between the two groups occurred (Figure 2).

Similar fragments from SW480 xenografts resulting from cells co-injected with matrigel, were washed in PBS and implanted into one group of five mice. No growth of the implanted fragments occurred.

*Effect of matrigel concentration on tumour growth*

A549 cells were co-injected with a range of matrigel concentrations from 12.1 to 1.8 mg $ml^{-1}$ into groups each of 5 mice over two experiments. At a concentration of 1.8 mg $ml^{-1}$ matrigel, there was no improvement in growth rate compared to controls. However at 3.6 mg $ml^{-1}$ growth rate was significantly increased ($P < 0.01$). Growth rate continued to increase with increasing matrigel concentration, up to the highest concentration attainable of 12.1 mg $ml^{-1}$ (Figure 3).

*Effect of matrigel components on xenograft growth, tumourigenicity and histology*

WiDr cells were co-injected sc into groups each of five mice with either purified laminin, laminin with entactin, matrigel, or medium only. The use of either matrigel or purified laminin resulted in a significant increase in final mean

**Figure 1** Effect of matrigel on tumour growth of a panel of four cell lines in nude mice, following injection of cells in 0.1 ml 10 mg ml$^{-1}$ matrigel (-●-) or complete medium (-+-). **a**, $1 \times 10^6$ A549, $P<0.02$, **b**, $1 \times 10^7$ SW480, $P<0.02$, **c**, $1 \times 10^6$ WiDr $P<0.001$, **d**, $1 \times 10^6$ HT1080scc2 $P<0.1$. Tumourigenicity was in the range 90–100% with the exception of HT1080scc2 cells injected in medium only (50%). SW480 tumours resulting from initial inoculations without matrigel regressed from 100% to 30% tumourigenicity by day 41.

tumour volume compared to that seen when cells were injected in medium only, whereas laminin with entactin had no such effect (Figure 4). Histological examination of the tumours was performed. The control tumours exhibited monomorphic epithelial-like cells with prominent nucleoli, arranged in closely packed 'bundles', with a high mitotic index. The centres of the tumours were highly necrotic. Tumours from both the purified laminin and the laminin/entactin groups were essentially the same as the controls, the stroma between the tumour bundles possibly being more prominent. Tumours derived from cell suspensions injected with matrigel however, had a tendency towards a more 'adenoid' and less solid appearance (Figure 5).

Similar results were obtained when A549 cells were used (not shown). Co-injection of A549 cells with collagen IV, or a mixture of collagen IV and laminin/entactin did not produce any increase in tumour growth. A further group of five mice were given A549 cells co-injected in a weak fibrin clot, produced by the catalytic conversion of fibrinogen to fibrin by the addition of thrombin, which thus acted as a non-matrigel derived substrate control. The use of fibrin did not increase xenograft growth relative to control growth rates (data not shown).

*Growth of orthotopic tumours by co-injection of SW480 cells with matrigel*

A group of five mice was injected intracaecally with a suspension of SW480 cells in 10 mg ml$^{-1}$ matrigel. A second group was injected in a similar manner with cells in PBS only. After 8 weeks post implantation the non-matrigel group had not developed any visible tumours, whereas 100% of the group of animals given cells with matrigel had developed small tumours with a mean of $30.4 \pm 10.1$ (standard error) mg in the caecal wall. There were no signs of macro- or micrometastases in the liver, lungs nor spleen of any of the tumour-bearing animals.

### Discussion

We have demonstrated the tumour-enhancing property of matrigel for the panel of four cell lines, selected in this study according to their differing growth characteristics *in vivo*. Tumour incidence in all four cell lines was either increased or maintained at 100% whilst the latency period was reduced. Tumour growth rate increased in three cell ines. Matrigel was

**Figure 2** Effect of subsequent passage on A549 xenografts grown with (-●-) or without (-+-) matrigel. $P > 0.1$.

**Figure 4** Xenograft growth in nude mice following co-injection of $1 \times 10^6$ WiDr cells with matrigel (-●-), purified laminin (-X-), laminin with entactin (-O-) or medium only (-+-). Both matrigel ($P < 0.001$) and purified laminin ($P < 0.02$) significantly increased tumour growth. 100% tumorigenicity (5 animals/group).

**Figure 3** Effect of matrigel concentration on the growth of A549 cells in nude mice. $1 \times 10^6$ cells were co-injected in 0.1 ml matrigel at 12.1 mg ml$^{-1}$ (-■-) $P < 0.001$, 6.1 mg ml$^{-1}$ (-Δ-) $P < 0.01$, 3.57 mg ml$^{-1}$ (-□-) $P < 0.01$, 1.76 mg ml$^{-1}$ (-▲-) or medium only (-+-). 5 animals/group. Tumourigenicity 80–100%.

therefore able to enhance the growth of tumours derived from cell lines which exhibit either long (A549 and SW480), or short (WiDr), tumour doubling times. In the light of these results, we see no obvious reason why matrigel failed to facilitate an increase in growth rate of the HT1080scc2 derived tumours. However the latter is the only cell line in the panel which is fibroblastic in origin.

Published observations have shown that when small cell lung carcinoma cells, isolated from tumours previously induced by co-injection with matrigel, are implanted sc, tumours develop only if these cells are once more transplanted in matrigel (Fridman *et al.*, 1991). We report that when fragments of adenocarcinoma A549 tumours are passaged directly into nude mice, the increased growth rate of tumours derived from cells co-injected with matrigel is not maintained. This strongly suggests that the action of matrigel in tumour growth promotion may not be related to the selection of a more tumourigenic sub-population of cells, but to the creation of a more favourable environment for growth.

Examination of stained sections of WiDr and A549 tumours showed that, when grown in the presence of matrigel, the cells formed a more differentiated tissue, piling up around what appeared to be central lumen-like structures (Figure 5). A similar histological appearance has been reported for Walker-256 murine breast carcinosarcoma cells in tumours derived from co-injection with matrigel (Vukicevic *et al.*, 1992). No other morphological changes were observed, apart from those related to the increase in tumour volume over the control tumours, such as larger areas of central necrosis and increased vascularisation. Similar growth characteristics were observed with SW480 cells implanted intracaecally, where, although an improvement in tumour growth occurred after co-injection with matrigel, tumour morphology was identical to that observed in matrigel-induced sc tumours of this cell line and there were no observable micro-metastases in the liver or spleen of the host mice. We recognise that this work concerned with the effects of matrigel on tumour growth at the orthotopic site is of a preliminary nature, but we believe it is the first indication of a possible use for basement membrane products as substrates for improved orthotopic models of cancer. We have also observed that improvement in xenograft growth rate is proportional to Matrigel concentration. This confirms the finding of Fridman *et al.* (1991) with regard to the dose-dependent effects of Matrigel on tumour growth, where doses

**Figure 5** Morphology of WiDr adenocarcinoma cells grown with matrigel. Cells formed solid tumours when injected sc in nude mice (**a** & **b**). Co-injection with matrigel resulted in a more glandular morphology (**c** & **d**). Bar = 18 μm.

of matrigel as low as 0.2 mg ml$^{-1}$ Matrigel affected tumour growth of B16F10 melanoma cells. In the present study, concentrations at least 15 fold higher than this were required to influence the growth of A549 tumours, a result probably relating to the different growth characteristics of these two cell lines.

Growth factors present in entire matrigel may be partly responsible for these concentration-dependent mitogenic effects, however it has been observed that these effects also occur when growth factor free matrigel is used (Fridman *et al.*, 1991). One possible interpretation of these observations is that the multi-layered basement membrane-like structure of the gel, formed by matrigel on reconstitution at 37°C, may facilitate tumour growth by holding the cells together and allowing a greater influence on cell growth by autocrine factors. It has previously been shown that a fibrin clot can protect human tumour cells from the cytotoxic activity of natural killer (NK) cells *in vitro* (Gunji *et al.*, 1988). It is possible that matrigel may protect implanted cells from the NK cells present in athymic mice. However, co-injection of A549 cells in a fibrin-thrombin coagulate did not result in any improvement in tumour growth over cells injected with medium only. This suggests that factors other than cellular protection and initiation of aggregation are involved.

Collagen IV is important in the structural formation of basement membrane architecture. It forms a highly cross-linked scaffolding of chords, and in its intact triple-helical conformation it anchors the laminin/entactin complex, aiding cell binding (Martin & Timpl, 1987; Timpl, 1989). We found that when A549 cells were co-injected with collagen type IV, tumour growth was not increased, suggesting that basement membrane proteins have specific roles in tumour growth.

Laminin is bound in a high affinity equimolar ratio to entactin which mediates the binding of laminin to collagen IV, whereas purified laminin shows no such binding (Aumailley *et al.*, 1989). We have found that co-injection of purified, entactin-free, laminin significantly increases the growth of A549 and WiDr tumours, whereas laminin plus entactin has no such effect (Figure 4). The reasons for these results are unknown. Previous reports of co-injection with purified laminin have indicated that in most cases it does not enhance tumour growth, and it has been suggested that this is because of the highly soluble nature of laminin which renders it susceptible to degradation *in vivo* (Fridman *et al.*, 1990). However, purified laminin has been shown to stimulate cell proliferation in established cell lines. This activity appears to originate in domain 1 of the moleculer which is rich in EGF-like repeats, and it has been suggested that this part of the molecule may be accessible to cells only during early stages of tissue development or following injury, where the basement membrane is either not intact or has been damaged (Panayotou *et al.*, 1989). Thus the co-injection of cells with purified laminin may imitate this situation, causing increasing cell growth, although the situation is a complex one and other factors such as interaction with host proteins could be involved.

Our studies indicate that reconstituted basement membrane can form a basis for improved *in vivo* models of tumour growth using cell lines with varied growth characteristics. Assay systems could be established which utilise reduced group sizes, greater use of the orthotopic site and relatively small amounts of matrigel. We have shown that laminin plays an important part in the growth stimulatory effects of matrigel *in vivo*, thus confirming similar effects already reported for this protein *in vitro*.

We are grateful to Mrs C.P.H. Willmott and Miss J.M. Sanderson for their expert technical assistance during the course of this study, to Miss A. Rowlands for discussions on the histology and to Dr K. Affleck and Dr J.E. Beesley for their careful reading and correcting of the manuscript.

### References

AUMAILLEY, M., WIEDEMANN, H., MANN, K. & TIMPL, R. (1989). Binding of nidogen and the laminin-nidogen complex to basement membrane collagen type IV. *Eur. J. Biochem.*, **184**, 241-248.

BARSKY, S.H., RAO, C.N., WILLIAMS, J.E. & LIOTTA, L.A. (1984). Laminin molecular domains which alter metastasis in a murine model. *J. Clin. Invest.*, **74**, 843-848.

DYKES, D.J., MAYO, J.G., ABBOTT, B.J., HARRISON, S.D. Jr, LASTER, W.R., SIMPSON-HERREN, L., GRISWOLD, D.P. & BOYD, M.R. (1989). *In vivo* growth characteristics of human tumour xenografts from the CIN *in vitro* 'disease-oriented' drug discovery program. *Proc. Am. Assoc. Cancer Res.*, **30**, 614.

FRIDMAN, R., GIACONNE, G., KANEMOTO, T., MARTIN, G.R., GAZDAR, A.F. & MULSHINE, J.L. (1990). Reconstituted basement membrane (matrigel) and laminin can enhance the tumourigenicity and the drug resistance of small cell lung cancer cell lines. *Proc. Natl Acad. Sci. USA*, **87**, 6698-6702.

FRIDMAN, R., KIBBEY, M.C., ROYCE, L.S., ZAIN, M., SWEENEY, T.M., JICHA, D.L., YANNELLI, J.R., MARTIN, G.R. & KLEINMAN, H.K. (1991). Enhanced tumour growth of both primary and established human and murine tumour cells in athymic mice after coinjection with matrigel. *JNCI*, **83** (11), 769-774.

FRIDMAN, R., SWEENEY, T.M., ZAIN, M., MARTIN, G.R. & KLEINMAN, H.K. (1992). Malignant transformation of NIH-3T3 cells after subcutaneous co-injection with a reconstituted basement membrane (matrigel). *Int. J. Cancer*, **51**, 740-744.

GIAVAZZI, R., CAMPBELL, D.E., JESSUP, J.M., CLEARY, K. & FIDLER, I.J. (1986). Metastatic behaviour of tumour cells isolated from primary and metastatic human colorectal carcinomas implanted into different sites in nude mice. *Cancer Res.*, **46**, 1928-1933.

GUNJI, Y. & GORELIK, E. (1988). Role of fibrin coagulation in protection of murine tumour cells from destruction by cytotoxic cells. *Cancer Res.*, **48**, 5216-5221.

LIN, J.-C., CHENG, J.-Y., TZENG, C.-C., YEH, M.-Y. & MENG, C.-L. (1991). An animal model for colon cancer metastatic cell line with enhanced metastasizing ability: establishment and characterisation. *Dis. Colon Rectum*, **34**, 458-463.

LIOTTA, L.A., RAO, C.N. & WEWER, U.M. (1986). Biochemical interactions of tumour cells with the basement membrane. *Ann. Rev. Biochem.*, **55**, 1037-1057.

MARTIN, G.R. & TIMPL, R. (1987). Laminin and other basement membrane components. *Ann. Rev. Cell Biol.*, **3**, 57-85.

NOEL, A., SIMON, N., RAUS, J. & FOIDART, J.M. (1992). Basement membrane components (matrigel) promote the tumorigenicity of human breast adenocarcinoma MCF7 cells and provide an *in vivo* model to assess the responsiveness of cells to estrogen. *Biochem. Pharm.*, **43**, 1263-1267.

PANAYOTOU, G., END, P., AUMAILLEY, M., TIMPL, P. & JURGEN, E. (1989). Domains of laminin with growth-factor activity. *Cell*, **56**, 93-101.

PATERSON, H., REEVES, B., BROWN, R., HALL, A., FURTH, M., BOS, J., JONES, P. & MARSHALL, C. (1987). Activated N-*ras* controls the transformed phenotype of HT1080 human fibrosarcoma cells. *Cell*, **51**, 803-812.

PRETLOW, T.G, DELMORCO, C.M., DILLEY, G.G., SPADAFORA, C.G. & PRETLOW, T.P. (1991). Transplantation of human prostatic carcinoma into nude mice in matrigel. *Cancer Res.*, **51**, 3814-3817.

SORDAT, B. & WANG, H.K. (1984). Human colorectal tumour xenografts in nude mice: expression of malignancy. *Behring. Inst. Mitt.*, **74**, 291-300.

TIMPL, R. (1989). Structure and biological activity of basement membrane proteins. *Eur. J. Biochem.*, **180**, 487-502.

TRAINER, D.L., KLINE, T., McCABE, F.L., FAUCETTE, L.F., FEILD, J., CHAIKIN, M., ANZANO, M., RIEMAN, D., HOFFSTEIN, S., LI, D.-J., GENNARO, D., BUSCARINO, C., LYNCH, M., POSTE, G. & GREIG, R. (1988). Biological characterisation and oncogene expression in human colorectal carcinoma cell lines. *Int. J. Cancer*, **41**, 287-296.

VUKICEVIC, S., SOMOGYI, L., MARTINOVIC, I., ZIC, R., KLEINMAN, H.K. & MARUSIC, M. (1992). Reconstituted basement membrane (matrigel) promotes the survival and influences the growth of murine tumours. *Int. J. Cancer*, **50**, 791-795.

WEST, M.L., KENG, P.C., SIEDMANN, D.W. & SUTHERLAND, R.M. (1987). A human colon adenocarcinoma xenograft - radiation response, cellular composition, and tumour disaggregation. *JNCI*, **78**, 371-376.

WORKMAN, P., BALMAIN, A., HICKMAN, J.A., McNALLY, N.J., MITCHISON, N.A., PIERREPOINT, C.G., ROWLATT, C., STEPHENS, T.C. & WALLACE, J. (1988). UKCCCR guidelines for the welfare of animals in experimental neoplasia. *Br. J. Cancer*, **58**, 109-113.

YURCHENKO, P.D. & SCHITTNY, J.C. (1990). Molecular architecture of basement membranes. *FASEB J.*, **4**, 1577-1590.